# Referral uptake after diabetic retinopathy screening with artificial intelligence-assisted care pathways: a systematic review and meta-analysis

**DOI:** 10.1038/s41746-026-02616-3

**Published:** 2026-04-17

**Authors:** James A. Leigh, Alex Sherrington, Angus R. J. Barber, Angus W. Turner, Michael Kidd, John Powell, Catherine Pope

**Affiliations:** 1 Nuffield Department of Primary Care Health Sciences, University of Oxford, Oxford, UK; 2 Lions Eye Institute, Lions Outback Vision, Nedlands, WA Australia; 3 Centre for Ophthalmology and Visual Science, The University of Western Australia, Crawley, WA Australia; 4 School of Biomedical Sciences, University of Western Australia, Crawley, WA Australia; 5 International Centre for Future Health Systems, University of New South Wales, Kensington, NSW Australia

**Keywords:** Computational biology and bioinformatics, Diseases, Health care, Medical research

## Abstract

Artificial intelligence (AI) is an accurate screening tool for diabetic retinopathy (DR), the leading cause of blindness among working-aged adults. However, its impact on referral uptake is uncertain. We searched Embase, MEDLINE, Scopus, Web of Science and Cochrane Library databases from year 2000 to February 17, 2025. Randomised and non-randomised studies comparing referral uptake after AI-assisted DR screening versus standard of care were included. 2644 articles were identified, and six included for analysis. The relative risk of DR referral uptake with AI-assisted screening compared with the status quo was 1.89 (95% CI, 1.18, 3.03, *I*^*2*^ = 91.9%). Settings which underwent referral pathway transformation from routine to targeted referrals for DR demonstrated the greatest effect size. Most (*n* = 4) studies also utilised behavioural change interventions enabled by immediate results acquisition of AI to enhance health-seeking behaviour. Our findings suggest the effectiveness of DR screening is derived not only from diagnostic technology, but from AI-enabled care pathway redesign encompassing both health system transformation and coordinated patient-facing interventions which improve referral uptake.

## Introduction

Diabetic retinopathy (DR) is the leading cause of blindness among working-aged adults and early detection is critical for timely management to prevent vision loss^1,2^. If left untreated, DR can progress to vision-threatening DR, proliferative DR and/or diabetic macular oedema, the latter of which can occur at any stage of disease^3^. The magnitude of diabetes and its sequelae including DR require large-scale screening to enable early intervention, as described in the recent Lancet Global Health Commission on Global Eye Health and contemporary modelling of diabetes prevalence worldwide^1,4^.

Multiple artificial intelligence (AI) algorithms have been developed and validated for DR^5–8^ screening and it is the most frequently implemented deep learning application in prospective real-world studies^9^. Several studies have reported its potential in addressing eye health inequities through improved access to screening in under-resourced areas and/or servicing priority populations. It is also considered a cost-effective intervention across multiple healthcare contexts. However, these economic modelling studies are limited by a lack of real-world data to inform parameter selection including transition probabilities, utility values, regional prevalence and uptake of screening and follow-up^10^. Indeed, beyond challenges of access and attendance at routine DR screening, referral uptake for those with referable DR is sub-optimal^11,12^ resulting in unmanaged patients at risk of blindness.

Real-world implementation of DR screening with AI-assisted care pathways has accelerated globally since 2020^8,13–22^, yet evidence on clinical and health system outcomes such as referral uptake remains limited. A recent meta-analysis of four studies^13–15,17^ by Rahmati et al.^23^ showed positive effects of AI-assisted DR screening on referral uptake, but did not explore mechanisms underpinning these differences.

A contemporary analysis of real-world AI-assisted DR screening studies which integrates behavioural and implementation science frameworks is timely to inform care pathway transformation and optimise clinical and cost-effectiveness. Our systematic review provides an updated meta-analysis of referral uptake after AI-assisted DR screening versus traditional referral pathways. We defined referral uptake as attendance at a scheduled follow-up appointment after undergoing DR screening. Our review also explored under what conditions and through what mechanisms AI-assisted screening workflows might improve rates of referral uptake for DR.

## Results

### Study selection

**Fig. 1:**
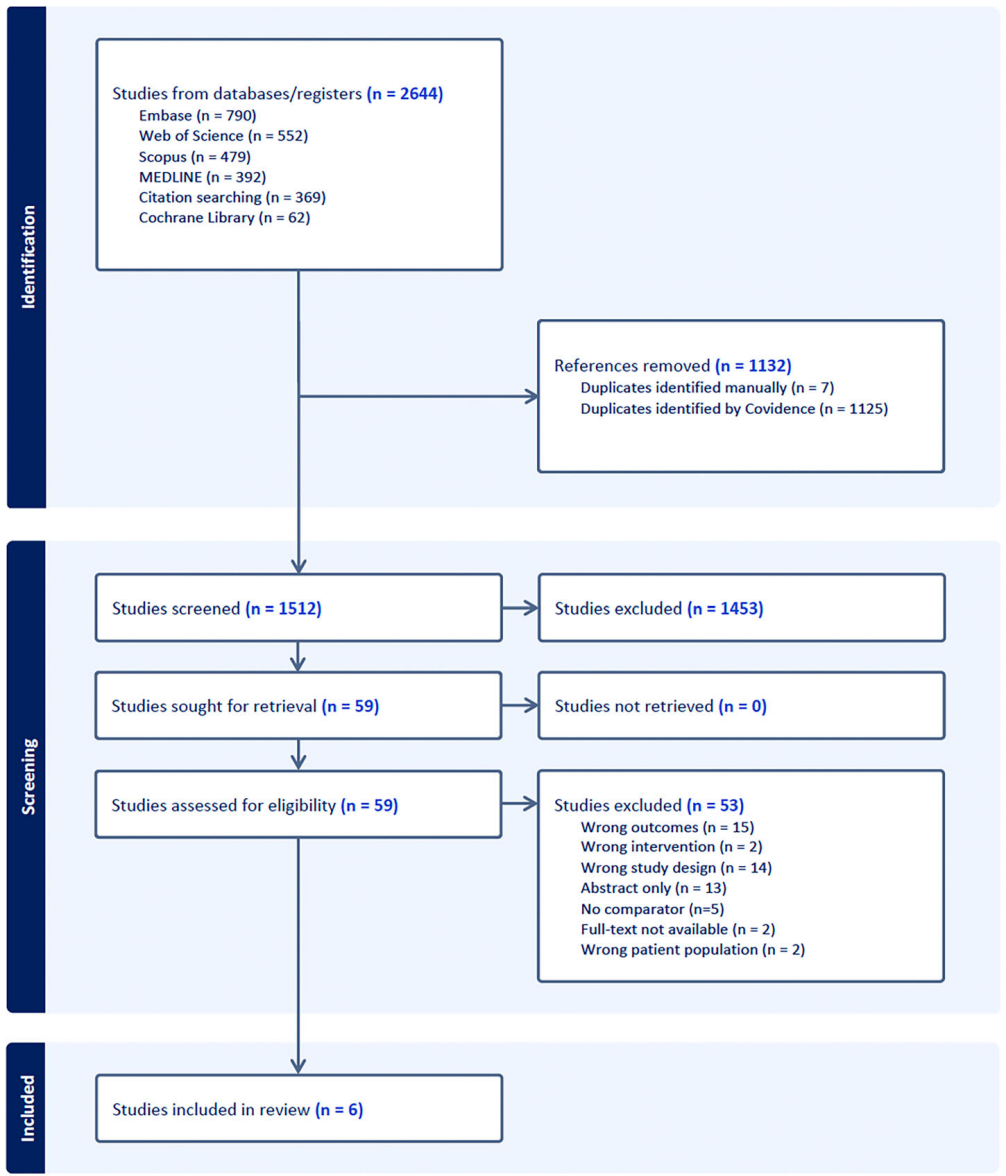
PRISMA diagram showing study selection process.

From 2644 articles identified across five databases (Embase *n* = 790, MEDLINE *n* = 392, Web of Science *n* = 552, Scopus *n* = 479, Cochrane Library *n* = 62, citation searching *n* = 369), 1512 remained after duplicate removal. Title and abstract screening excluded 1453 irrelevant articles, leaving 59 for full-text review. Following detailed assessment, 53 articles were excluded: wrong outcome (*n* = 15), wrong study design (*n* = 14), abstract only (*n* = 13), no comparator (*n* = 5) wrong intervention (*n* = 2), wrong patient population (*n* = 2), and unavailable full text article (*n* = 2). Six studies met final inclusion criteria. A summary of our process for study selection is described in the PRISMA diagram (Fig. 1).

### Study characteristics

Among our six included studies, three were conducted in the USA^13,15,17^, and one in each of Rwanda^14^, Thailand^16^, and China^18^. Study designs varied, including RCTs (*n* = 2)^13,14^, prospective cohort studies (*n* = 3)^16–18^, and a retrospective cohort study (*n* = 1)^15^. The RCT by Wolf et al. compared AI-assisted screening with routine referrals for youth with diabetes^13^, whereas Mathenge et al. used AI to determine the referral eligibility for all participants, randomising those with referable DR to receive immediate feedback of AI grading at the point-of-care (intervention) or delayed communication of referral advice after human grading was completed 3–5 days later (comparator)^14^. This approach sought to isolate the effect of result timeliness on referral uptake^14^. For prospective cohort studies, Chotcomwongse et al. used AI and manual grading on alternate weeks^16^; Li et al. compared an initial six weeks of manual grading followed by six weeks of AI-assisted screening^18^; and Liu et al. compared AI referral uptake with a historical dataset of consecutive patients routinely referred for DR^17^. Dow et al., a retrospective cohort study, compared an initial 18 months of manual grading via teleophthalmology before AI’s implementation^15^. An overview of the characteristics for each of the included studies is provided in Table 1. Studies are characterised by the author, journal and year of publication, country, study design, sample size, follow-up period, definition of referable DR and referral uptake (%).

**Table 1:** Study characteristics

Author (citation), journal, year	Country	Study design	Sample size (AI/control)	Follow-up period	Referable DR definition (ICDR)	Referral Uptake (AI/control)
Wolf et al.^13^, Nature Communications, 2024	USA	Parallel group randomised controlled trial	25/83	6 months	≥Moderate NPDR, DME	64%/21.7% **(**+**42.3%)**
Mathenge et al.^14^, Ophthalmology Science, 2022	Rwanda	Parallel group randomised controlled trial	136/139	12 months	≥Moderate NPDR	51.5%/39.6% **(**+**11.9%)**
Li et al.^18^, Nature Medicine, 2024	China	Prospective cohort study, two-arm sequential	144/154	2 weeks	≥Moderate NPDR, DME	77.8%/58.4% **(**+**19.3%)**
Liu et al.^17^, Ophthalmology Retina, 2021	USA	Prospective cohort study with historical comparator	92/974	12 months	≥Moderate NPDR, DME, ungradable	55.4%/18.7% **(**+**36.7%)**
Dow et al.^15^, Clinical Ophthalmology, 2023	USA	Retrospective cohort study	279/117	90 days	≥Moderate NPDR, Ungradable (if in the hybrid workflow)	35.5%/12.0% (+**23.5%)**
Chotcomwongse et al.^16^, Ophthalmology and Therapy, 2025	Thailand	Prospective cohort study, two-arm alternating	129/175	Unspecified	≥Severe NPDR, DME, Ungradable	89.1%/70.9% **(**+**18.3%)**

Bold values: Risk difference for referral uptake (%).

### Results of syntheses

**Fig. 2:**
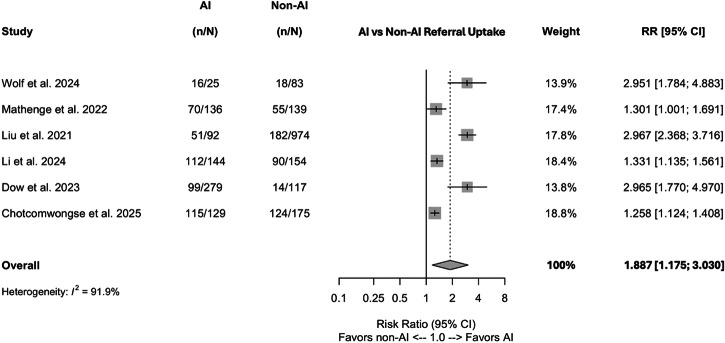
Forest plot of relative risk of AI-assisted DR screening on referral uptake.

**Fig. 3:**
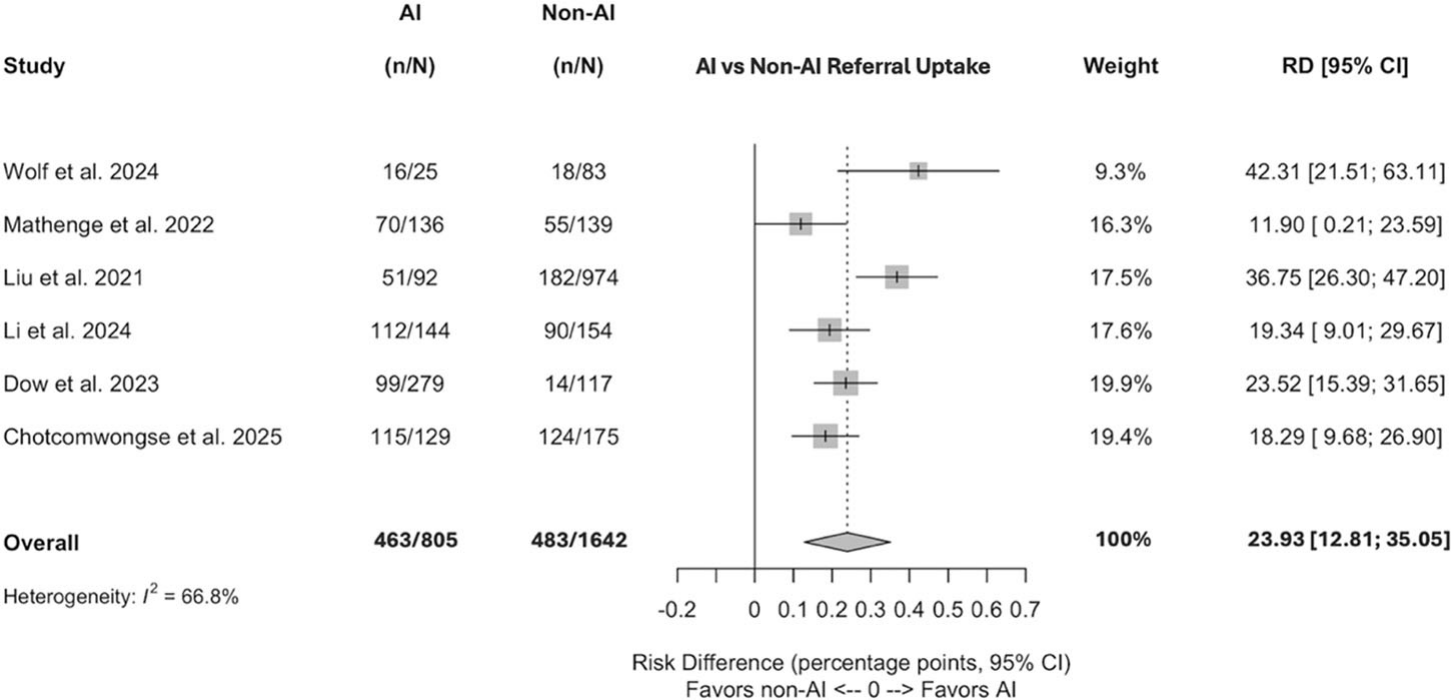
Forest plot of risk difference of AI-assisted DR screening on referral uptake.

Based on the random effects meta-analysis of six studies comparing AI-assisted versus non-AI referrals for DR, the pooled results suggest that AI screening improves referral uptake compared to traditional care pathways. It should be noted there were two distinct, status quo care pathways: studies providing routine referrals for people with diabetes without prior screening^13,17^ and manual grading workflows including asynchronous teleophthalmology^14–16,18^. We calculated a relative risk (RR) of 1.887, 95% CI, 1.175, 3.030 (Fig. 2) and risk difference (RD) of 23.93%, 95% CI, 12.81%, 35.05% (Fig. 3) for referral uptake. However, our analysis showed substantial heterogeneity between studies (high I² value, 91.9%), suggesting that the effectiveness of AI versus non-AI referrals may depend on specific contextual factors such as healthcare setting, patient population, follow-up duration, and implementation approach. The clinical significance of these findings supports the potential for AI-assisted DR screening to not only augment diagnostic accuracy at the point of referral but also enhance clinical outcomes through improved referral uptake.

### Risk of bias

Overall, the risk of bias among studies was low to serious. Both RCTs by Mathenge et al.^14^ and Wolf et al.^13^ were deemed low risk of bias as per the RoB-2. For observational studies assessed with ROBINS-I, Li et al.^18^ showed the most robust quality with low-to-moderate risk across domains, limited primarily by lack of sensitivity analyses. Liu et al.^17^, Dow et al.^15^, and Chotcomwongse et al.^16^ demonstrated serious risk of bias due to confounding from co-interventions (automated reminders^16^, proactive scheduling calls^17^), sequential implementation with potential for temporal confounding^15,17^, and fundamentally different comparators (AI-targeted versus routine referrals)^17^. None of the observational studies adequately controlled for confounding variables. Detailed risk of bias assessments are presented in Supplementary Tables 5 and 6.

### Results of individual studies

We analysed studies’ data on comparative referral uptake according to intervention type, including use of co-interventions, comparator approach, and baseline rates of referral uptake to better understand the contexts in which AI-enabled workflows may be most effective.

Two studies compared AI-assisted targeted referrals to routine referral strategies where all diabetic patients were referred without results. Wolf et al.‘s ACCESS RCT (*n* = 108) in a paediatric diabetes centre found AI-assisted screening with targeted referrals achieved 64% uptake (16/25 referrals) versus 22% (18/83) with routine eye care provider referrals, representing a + 42% absolute difference in uptake (95% CI: +21% to +63%, *p* < 0.001)^13^. Liu et al.‘s prospective cohort study (*n* = 1066) comparing AI screening to historical routine referral controls demonstrated 55.4% uptake at 12 months versus 18.7% in controls (+36.7%, *p* < 0.0001), though this included proactive scheduling calls as a co-intervention^17^.

Four studies compared AI-assisted screening directly to manual grading workflows. Mathenge et al.‘s RAIDERS RCT (*n* = 275) uniquely isolated the effect of immediate versus delayed feedback by randomising patients to immediate AI result acquisition with a colour-coded report based upon severity, or delayed manual grading (3–5 days) with SMS and phone calls to flag results, finding 51.5% versus 39.6% referral uptake respectively ( + 11.9%, OR 1.62, 95% CI: 1.00–2.61, *p* = 0.048)^14^. Li et al.‘s sequential cohort study (*n* = 298) compared AI-assisted screening with treatment advice based upon multimodal patient data (medical history, physical examinations, laboratory tests) and retinal images, to unassisted manual grading, achieving 77.8% versus 58.4% referral uptake within two weeks (+19.4%, *p* = 0.001)^18^. Chotcomwongse et al.‘s alternating-week study design (*n* = 304) compared AI screening coupled with automated text reminders before scheduled follow-up to nurse-led manual grading, finding 89.1% versus 77.3% referral uptake (+11.8%, *p* = 0.158, not statistically significant)^16^. Dow et al.‘s retrospective study (*n* = 396) showed 35.5% referral uptake with AI workflow versus 12.0% with manual grading when restricting analysis to university eye institute appointments (+23.5%, *p* = 0.000004)^15^.

A clear pattern emerged whereby studies with lower baseline rates of referral uptake demonstrated larger absolute improvements. Studies comparing AI to routine referral approaches with typically lower baseline rates of uptake (18.7–22.0%) showed the largest effect sizes (+36.7% to +42.0%)^13,17^, while studies comparing AI to existing manual screening workflows with higher baseline rates of uptake (39.6–77.3%) showed more modest improvements (+11.8% to +19.4%)^14,16,18^. This suggests AI-assisted workflows may have greatest impact in settings with currently poor rates of referral uptake, particularly where routine referral strategies are being replaced by targeted, screening-based approaches to DR referral supported by AI.

Attribution of improvements in completed referrals specifically to AI versus other workflow components remains challenging given the frequent inclusion of co-interventions and fundamental differences in referral strategies across studies. Several studies included co-interventions alongside AI-assisted DR screening with variable effectiveness^13,16–18^. Rates of referral uptake across these studies ranged from 55.4%^17^ to 89.1%^16^ and effect sizes ranged from +11.8%^16^ to +42.0%^13^.

Chotcomwongse et al.’s AI platform provided automated text message reminders to patients with referable DR several days before their scheduled referral appointment^16^. Evidence suggests the use of text reminders positively influence rates of referral uptake and this may enhance the impact of AI screening, thereby exaggerating effectiveness^24^. Similarly, Liu et al. conducted a scheduling phone call within two weeks of the AI screening appointment to arrange a referral appointment^17^. Scripted education after screening was provided to intervention and comparator groups in the ACCESS RCT by Wolf et al.^13^. Li et al. provided patients with an AI generated screening report providing treatment advice which we classed as education^18^.

## Discussion

We identified several patterns linking effect sizes to health system context, intervention design, and co-interventions. In our discussion we will explore the relationships between studies through a patient-level lens and health systems lens^25,26^. The Behaviour Change Wheel framework describes how interventions affect behaviour through three mechanisms: capability, opportunity, and motivation (COM-B system)^25^. The Non-adoption, Abandonment, and challenges to Scale-up, Spread and Sustainability (NASSS) Framework provides a comprehensive foundation for theorising how AI-assisted DR screening may support the uptake of referrals from a health system perspective^26^.

AI-assisted pathways improved absolute referral uptake by 23.9% overall (RR 1.89, 95% CI 1.18–3.03), reiterating Rahmati et al.‘s prior meta-analysis^23^ of four included studies^13–15,17^. In absolute terms, for approximately every four patients screened through an AI-assisted pathway compared with the status quo, one additional patient completed their referral. This is clinically significant given the progressive nature of DR and risk of blindness if left untreated through non-uptake of a referral. Despite the clinical significance, substantial heterogeneity (I² = 92%) indicates marked variability between studies and context-dependent effectiveness which will be discussed further.

Studies replacing routine referrals for all people with diabetes with AI-assisted targeted referrals in low-performing systems (baseline adherence 18.7–22.0%) achieved the largest improvements. Wolf et al. reported a + 42.0% improvement in a diabetology clinic screening youth with diabetes^13^, whilst Liu et al. reported a + 36.7% improvement in a primary care clinic screening adults with diabetes^17^. These low baseline rates of referral uptake align with other US reports showing 5–51% referral uptake within recommended timeframes^12^, indicating substantial potential benefit in these settings.

Conversely, studies comparing AI-assisted screening to existing manual grading workflows showed modest gains ranging from +11.8 to +23.5%^14–16,18^. This was most pronounced in in higher-performing systems (baseline referral uptake 39.6–77.3%)^14,16,18^ which indicates diminishing returns in settings with already functional screening infrastructure. Such a pattern suggests settings with routine DR referral strategies for all people with diabetes, or lacking systematic screening programmes, are likely to benefit most from implementing AI-assisted DR screening.

Timeframe of referral may also influence reported rates of uptake. Supplementary Table 9 provides a rearranged summary of findings table in descending order of timeframe for referral appointment. There was marked variation for timeframes among included studies ranging from 2 weeks for Li et al. in China^18^ up to 12 months for Liu et al. in the USA^17^. There was inconsistency in the effect of timeframe and rates of referral uptake. For example, Li et al., despite allowing just a fortnight for appointments reported referral uptake of 58.4% and 77.8% for comparator and intervention groups, respectively^18^. In contrast, Liu et al. recorded rates of referral uptake at 3 months, 6 months and 12 months from the date of screening and referral^17^. In this study there was a correlation between time and rates of uptake, increasing from 32.6% at 3 months to 55.4% at 12 months^17^. Such variability makes it difficult to ascertain the true effect of longer appointment timeframes on corresponding uptake. Future studies should therefore report rates of referral uptake at standardised intervals and describe the indication for these intervals e.g., urgent cases, non-urgent cases, or delayed follow-up within one year.

Based on evidence from comparative studies, AI-assisted DR screening is associated with increased referral uptake among patients with referable disease^13–18^. AI screening is associated with an approximately 12–42% increase in referral uptake compared to standard care^13–18^, with a RR of 1.89 (95% CI, 1.175, 3.030). However, these findings are supported by low-certainty evidence due to serious inconsistency in effect sizes across studies, heterogeneity (I^2^ = 92%) and potential publication bias. While all studies consistently demonstrate improved referral uptake after AI-assisted DR screening^13–18^, magnitude of benefit varies substantially from moderate improvements ( + 11.8%)^16^ to large effects ( + 42.0%)^13^.

We attribute substantial heterogeneity (I^2^ = 92%) to three principal sources. Firstly, comparator types whereby two studies replaced routine referrals with targeted, AI-assisted referrals^13,17^ showed greater effect sizes than those studies with a manual grading comparator^14–16,18^. Secondly, co-intervention usage varied across studies, with some utilising automated reminders^16^, scheduling calls^17^, or providing patient education alongside AI screening^13,18^. Thirdly, baseline health system performance appeared to influence the marginal gains, following a law of diminishing returns whereby settings with higher baseline rates of uptake experienced relatively lower effect size^14,16,18^, in contrast to lower-performing systems with greater margins for improvement^13,15,17^. These sources of heterogeneity will be explored in more detail and are summarised in Table 2.

**Table 2:** Intervention details for each included study, in descending order of risk of bias

Author (citation)	AI Algorithm	AI Output	Co-Intervention(s)	Referable DR Definition (ICDR)	% of Images Ungradable	Mydriasis (yes/no)	Retinal Camera
Wolf et al.^13^	IDx-DR (LumineticsCore™)	DED y/n, gradable y/n	Education about screening result	≥Moderate NPDR, DME	0%	No	Not discussed
Mathenge et al.^14^	Cybersight AI	DR referral y/n, macular anomaly y/n, vertical cup:disc ratio ≥0.7, gradable y/n	Nil	≥Moderate NPDR	Ungradable images excluded	Yes, if ungradable and pupil <2.5 mm	Topcon NW400
Li et al.^18^	DeepDR	DR severity, DME y/n, gradable y/n	Treatment advice for diabetes informed by LLM	≥Moderate NPDR, DME	Ungradable images excluded	Not discussed	Canon, Topcon, Carl Zeiss, Optomed, Microclear
Liu et al.^17^	EyeArt	DR severity, DME y/n, gradable y/n	Scheduling phone call within 2 weeks of screening	≥Moderate NPDR, DME, ungradable	29.4%	No	Canon CR-2
Chotcomwongse et al.^16^	ARDA	DR severity, DME y/n, gradable y/n	Automatic reminders sent to patient phone days before follow-up	≥Severe NPDR, DME, Ungradable	58.3%	No (Jan-Mar) Yes (Apr-Aug)	Topcon® TRC-NW400
Dow et al.^15^	IDx-DR (LumineticsCore™)	DR referral y/n, gradable y/n	Nil	≥Moderate NPDR, Ungradable (if in the hybrid workflow)	35.0%	No	Topcon NW400

AI-assisted screening appears to improve referral uptake through two mechanisms: patient-level behavioural interventions enabled by immediate results acquisition, and health system redesign by transforming referral pathways. It should be noted that immediate results acquisition is not unique to AI-assisted DR screening and could be provided through synchronous teleophthalmology or at the point-of-care by manual graders. However, findings from our review suggest the status quo for DR screening involves asynchronous teleophthalmology with delayed feedback of results^14–16,18^ or no prior screening as was the case in two North American studies by Wolf et al. and Liu et al.^13,17^. These findings reinforce the notion that whilst immediate feedback is possible with manual DR grading, the accessibility and democratisation of access to AI-assisted screeningmay favour its scale-up and spread in DR care pathways.

**Fig. 4:**
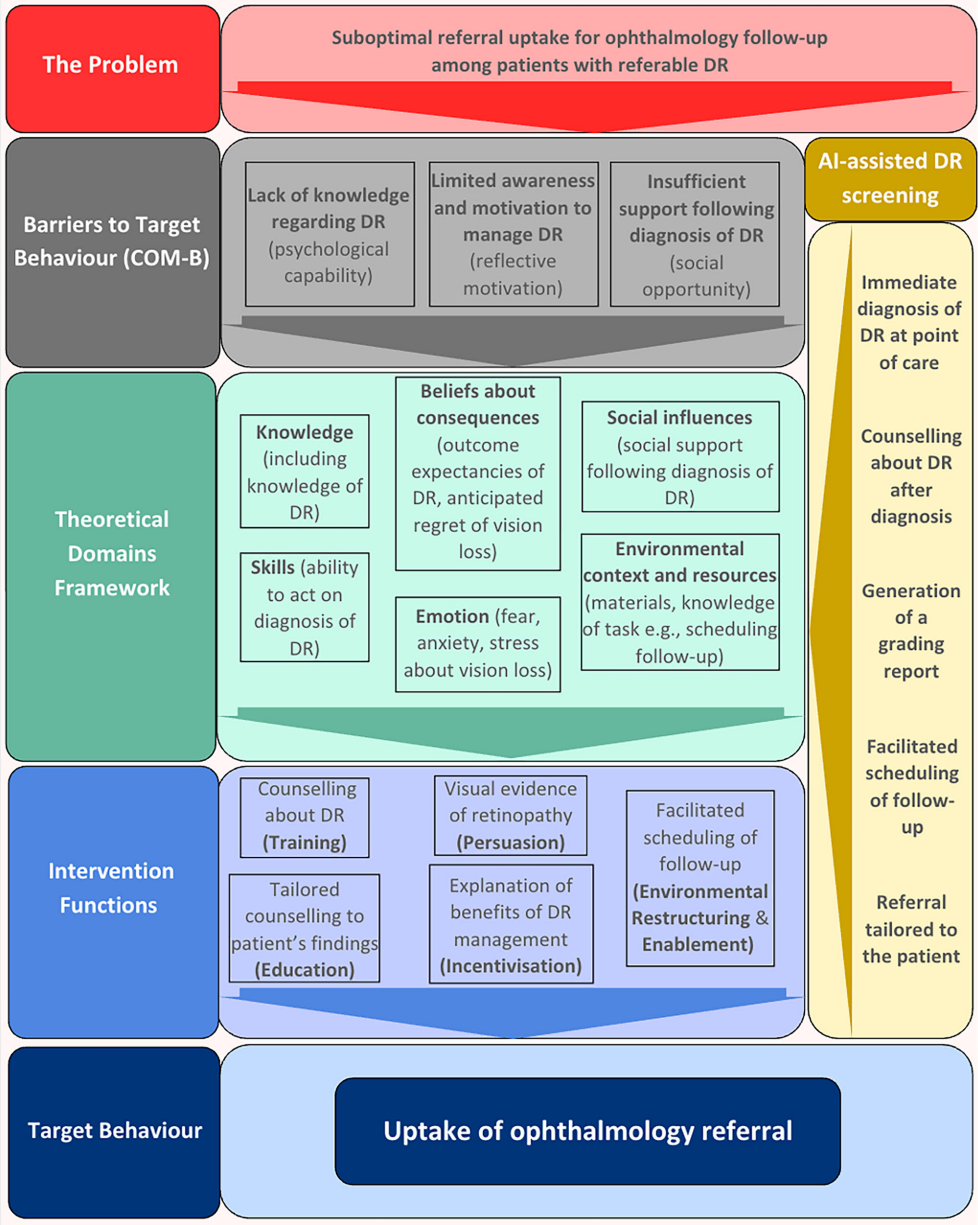
Logic model for the behavioural change due to AI-assisted DR screening.

Immediate AI results catalyse coordinated interventions across multiple behavioural domains^25^ (Fig. 4). Facilitated scheduling addresses *social opportunity* barriers^16,17^, point-of-care counselling enhances *psychological capability*^13^, and provision of personalised reports supports *reflective motivation*^18^. Mathenge et al.’s isolation of immediate versus delayed feedback after AI-assisted screening ( + 11.9% referral uptake) demonstrates that result timeliness alone modestly improves uptake through enhanced capability^14^. However, larger effect sizes in Wolf et al. ( + 42.0%)^13^ and Liu et al. ( + 36.7%)^17^ may reflect simultaneous targeting of multiple behaviour change domains, combining immediate feedback with scheduling support and patient education, or as a consequence of DR care pathway transformation. Figure 4 demonstrates our logic model for the theory underpinning patient-level behaviour change after AI-assisted DR screening and its co-interventions, enabled by immediate results at the point-of-care. The COM-B system is a framework proposing three key elements for behaviour change to occur through an intervention: capability, opportunity, and motivation^25^.

It should be noted Dow et al. achieved substantial improvement ( + 23.5%) without documented co-interventions for behaviour change^15^, suggesting workflow redesign alone may drive gains in referral uptake in some contexts, even with existing manual grading infrastructure such as asynchronous telehealth via remote grading centres. The relative contribution of AI diagnostic capability, immediate results acquisition, and enabled co-interventions, therefore remains uncertain.

AI’s effectiveness varies by organisational context, reflecting the NASSS Framework’s *value proposition* domain^26^. In settings where pre-existing workflows included routine referral strategies^13,17^, AI-assisted DR screening enabled transformation of care pathways by replacing routine referrals with targeted, evidence-based referrals. This system-level transformation yielded large effect sizes ( + 36.7–42.0% absolute improvement in referral uptake)^13,17^ thereby improving supply-side value.

Conversely, in settings with pre-existing manual grading workflows, AI-assisted DR screening provided incremental optimisation rather than transformation, yielding modest improvements ( + 11.8–23.5%)^14–16,18^ despite higher baseline performance (39–77%)^14,16,18^ save for Dow et al. (baseline of 12%)^15^. This pattern suggests AI’s value proposition from a health system perspective depends on whether existing care pathways require transformation or optimisation.

Our findings suggest the effectiveness of DR screening is derived not simply from diagnostic technology, but from AI-enabled care pathway redesign encompassing both health system transformation and coordinated patient-facing interventions which improve referral uptake.

Our findings have several clinical implications. AI-assisted screening pathways should be implemented comprehensively, not as isolated diagnostic tools. Settings likely to benefit most include those currently using routine referral strategies or lacking systematic screening infrastructure, where baseline rates of referral uptake are low (< 30%). Essential implementation components include immediate results delivery, point-of-care patient counselling, facilitated appointment scheduling, and provision of personalised grading reports.

From a policy perspective, reimbursement models should recognise that AI value derives from enabling workflow transformation rather than diagnostic substitution alone. Healthcare systems should budget for comprehensive pathway redesign, including staff training for patient-facing co-interventions, not merely AI software acquisition.

Future research priorities should include factorial randomised trials isolating contributions of AI diagnostic capability, immediacy of results acquisition, and specific co-interventions. Standardised approaches to managing ungradable images are also crucial to optimise referral efficiency by reducing false positive results masquerading as ungradable images. Critically, cost-effectiveness analyses across diverse health system contexts should integrate differential rates of referral uptake between AI and non-AI screening pathways as a parameter, or account for variability in this parameter through robust sensitivity analyses^10^.

This systematic review has several strengths. We conducted comprehensive searches across five databases, employed dual independent screening and risk of bias assessment, and included both randomised and observational studies reflecting real-world implementation contexts. Our integration of behavioural science theory (COM-B) and implementation science theory (NASSS) provides insights beyond meta-analysis of effect size. The included studies span diverse geographic settings (four continents) and health system contexts, supporting generalisability.

However, several limitations affect causal attribution. First, two studies compared AI-assisted targeted referrals to routine referral pathways for all people with diabetes^13,17^, in contrast to the remaining four studies whose comparators included manual grading via asynchronous teleophthalmology^14–16,18^. Such heterogeneity may limit the reliability of our random effects meta-analysis, the results of which we have sought to contextualise through a robust narrative synthesis. Second, temporal confounding affects retrospective studies using historical controls, where concurrent quality improvement initiatives or evolving health system factors may independently influence referral uptake. Third, co-interventions (facilitated follow-up scheduling, text reminders, patient education, grading reports) were implemented alongside AI in most studies^13,16–18^, precluding attribution of effects to specific pathway components.

Methodological heterogeneity further limits our synthesis. Referral appointment timeframes varied from two weeks^18^ to twelve months^17^ and definitions of referable DR differed. Pooling of randomised and non-randomised studies introduced complexity for interpretation which we sought to mitigate through design-appropriate risk of bias tools, applying GRADE to assess overall certainty, and conducting a robust narrative synthesis. Ungradable images were managed differently across studies, which warrants consideration as a potential source of bias. In principle, differential exclusion of ungradable images between AI and comparator groups could inflate referral uptake rates in one group relative to the other. However, among included studies in our review this risk was mitigated by design (Table 2). Three studies included ungradable images as referable DR, thus retaining them in the referral denominator^15–17^. Two studies excluded ungradable images from analysis entirely, prior to allocation to AI or comparator arms^14,18^. The final study by Wolf et al.^13^ reported rates of ungradable images of 0%, voiding this limitation.

These limitations undermine causal inference about AI’s isolated effect on referral uptake, but do not invalidate the central finding: comprehensive AI-assisted care pathways consistently improve DR referral uptake across diverse settings. The included populations represent typical diabetic screening cohorts in primary care, diabetology, and ophthalmology settings, supporting applicability to similar healthcare contexts internationally.

AI-assisted screening pathways consistently improve referral uptake among patients with referable DR across diverse settings, though low-certainty evidence limits attribution to specific pathway components. The largest improvements occur in settings replacing routine referrals with AI-assisted targeted referrals, while existing screening workflows show modest gains. Effective implementation requires comprehensive pathway redesign encompassing immediate results at the point of care, patient education, facilitated scheduling of follow-up, and personalised reports - not AI diagnostic software alone. Future research should include factorial trials to isolate specific contributions of AI capability, immediacy of results acquisition, and enabled co-interventions. Cost-effectiveness analyses should also integrate referral uptake parameters into modelling approaches.

## Methods

We conducted a systematic review and meta-analysis with narrative synthesis of studies reporting data on referral uptake after AI-assisted DR screening. Our review was conducted according to the Cochrane Library Guidelines^27^, Guidance on the Conduct of Narrative Synthesis in Systematic Reviews^28^, and reported according to the Preferred Reporting Items for Systematic reviews and Meta-Analyses (PRISMA) guidelines^29^. This study was registered with PROSPERO (registration number: CRD42025626427).

### Eligibility criteria

For inclusion in this review, studies must have reported rates of referral uptake among patients with referable DR; include AI-assisted DR screening as an intervention; and reported data on a comparator group without the use of AI. Both randomised and non-randomised study designs were eligible for inclusion, as pre-specified in our PROSPERO protocol, reflecting the limited number of RCTs evaluating AI-assisted DR screening pathways and the importance of capturing real-world implementation evidence. Articles were excluded if they did not explicitly study DR screening, did not use AI for image analysis in DR screening, did not report on rates of referral uptake, were not written in English and used an alternative study design to those previously mentioned. A summary of our eligibility criteria is provided in Supplementary Table 2.

### Information sources

We searched multiple online databases from year 2000 to February 17th, 2025, including Embase, MEDLINE, Web of Science, Scopus, and Cochrane Library; and accessed these databases through Ovid, Clarivate, Scopus and the Cochrane platforms.

### Search strategy

Searches were developed in collaboration with an Information Specialist. We used the following search in MEDLINE to demonstrate our strategy: “(Artificial Intelligence/OR Neural Networks, Computer/OR exp Machine Learning/OR Image Processing, Computer-Assisted/OR (Automated OR “autonomous AI” OR “computer based analysis” OR “convolutional neural network”).ti,ab.) AND (Treatment Adherence and Compliance/OR (Adherence OR “follow up” OR referral*).ti,ab.) AND (Diabetic Retinopathy/OR (“Diabetic eye*“ OR “diabetic retinopathy” OR “more than mild DR”).ti,ab.)”. A full description of our search strategy translated across Embase, MEDLINE, Scopus, Web of Science and the Cochrane Library is available in Supplementary Table 3. Corresponding authors were contacted for articles without full-text availability to request access.

### Selection process

Two independent reviewers (JL, AS) screened titles, abstracts and full text articles using Covidence^30^ with a third reviewer (AB) who settled disputes. Articles which met the eligibility criteria were identified for data extraction. Forward and backward citation searching was performed on included articles.

### Data collection process and data items

The outcomes for this systematic review were referral uptake among patients with referable DR after AI-assisted screening, compared with status quo referral pathways, and theoretical explanations for any differential uptake of referrals. Given the complexities of health system workflows, we extracted data on referral pathways including co-interventions which may have influenced rates of referral uptake, numbers of patients who were referred with and without AI, and corresponding numbers of patients who attended the referral. Other data items reported in Table 1 include study characteristics such as authors, journal and year of publication, the country and setting of each study, study design, timeframe for follow-up and target population. Table 2 reports intervention details for each study including the specific algorithm used for DR screening, the definition of referable DR, proportion of ungradable images and whether they were reported as referable DR, and use of mydriasis to address ungradable images due to small pupil size.

### Risk of bias assessment

Risk of bias and quality assessments were performed for included studies by JL and AS, using design-appropriate tools. The Cochrane Collaboration’s tool for assessing risk of bias in randomised trials (RoB-2)^31^ and ROBINS-I tools^32^ were used for RCTs and observational studies, respectively.

### Effect measures

For our primary outcome of referral uptake, we reported the intervention effect as absolute effect (risk difference [%]) and relative risk (RR) in the numbers of patients who completed follow-up for DR. We defined effect size categories as “not large” (RR between 0.5 and 2.0), “large” (RR < 0.5 or >2.0), or “very large” (RR < 0.2 or >5.0) for our certainty assessment.

### Synthesis methods

We conducted a random effects meta-analysis and narrative synthesis of included studies to explore both the effect size, and reasons for differential effect sizes across studies in different settings. Digital health interventions such as AI are inherently complex with heterogenous elements regarding integration into clinical workflows, algorithm outputs, and sociotechnical factors affecting real-world effectiveness in each setting^26^ which we aimed to interpret through included studies.

Our random effects meta-analysis utilised the Mantel-Haenszel method to pool risk ratios with 95% confidence intervals, implemented in RStudio 2025.05.0 + 496 using the *meta* package (version current as of analysis date [07/09/25]). The random effects model was selected due to clinical and methodological heterogeneity across studies. Statistical heterogeneity was assessed using I² statistic (with thresholds of <25% low, 25–50% moderate, 50–75% substantial, >75% considerable). Given the inclusion of only six studies, formal subgroup analyses and meta-regression were not performed. Cochrane guidance recommends a minimum of ten studies per covariate for meta-regression, and subgroup analyses with fewer than three studies per subgroup risk producing unreliable estimates^33^. Instead, we explored sources of heterogeneity qualitatively through structured narrative synthesis, examining patterns by comparator type, baseline referral uptake rate, and co-intervention intensity.

Our approach to narrative synthesis followed the “Guidance on the Conduct of Narrative Synthesis in Systematic Reviews”^28^. This guidance document outlines four steps to narrative synthesis: developing a theory of how an intervention works, why and for whom; developing a preliminary synthesis of findings of included studies; exploring relationships in the data; and assessing the robustness of the synthesis^28^. We developed a “theory of change”, as described by Weiss^34^, to inform our review, drawing on the Behaviour Change Wheel^25^ and Non-adoption, Abandonment, and challenges to Scale-up, Spread, Sustainability (NASSS) Frameworks^26^. These two frameworks were used to generate ideas regarding the reasons AI may change referral uptake for DR.

### Certainty of evidence

In line with the Cochrane Library Guidelines^27^ we used the Grading of Recommendations, Assessment, Development, and Evaluation (GRADE) approach and Guideline Development Tool^35^ for assessing the certainty of evidence^36^. There was no funding source for this study.

### Data sharing

Eligibility criteria, search strategies across selected databases, comparator details, risk of bias assessment criteria and results, and completed PRISMA Checklist are available in the supplementary material.

## Supplementary information

Supplementary information

Supplementary information
