# Supplementary material for: Referral uptake after diabetic retinopathy screening with artificial intelligence-assisted care pathways: a systematic review and meta-analysis
Source: NPJ Digit Med. 2026 Apr 17;9:468. doi: 10.1038/s41746-026-02616-3 (PMC13272687; doi:10.1038/s41746-026-02616-3)
Supplement: Supplementary file 1 — Supplementary information [file 41746_2026_2616_MOESM1_ESM.docx]

**Supplementary Material**

**Table of Contents**

*PICO Framework…………………………………………………………………….…2*

*Eligibility Criteria ………………………………………………………………………2*

*Search Strategy…………………………………………………………………………..3*

*Intervention Details ..…………………………………………………………….…….4*

*Comparator Details ……………………………………………………………………4*

*RoB-2 ……………………………………………………………………......................5*

*ROBINS-I………………………………………………………………………………..5*

*Summary of Findings………………………………………………………………….5*

*Co-interventions………………………………………………………………………..6*

*Referral Uptake & Timeframe………………………………………………………..6*

*Excluded Studies (Full-Text) ……………………………………………….………7-9*

*Extended Discussion………………………………………………………………..9-10*

**Supplementary Table 1: PICO framework applied to systematic review research question**

| **Population** | Patients with referable diabetic retinopathy |
| --- | --- |
| **Intervention** | AI-assisted screening for diabetic retinopathy prior to referral |
| **Comparator** | Referral without the use of prior AI screening |
| **Outcome** | Rates of adherence to eye care follow-up among patients with referable DR |

**Supplementary Table 2: Eligibility Criteria for Included Studies**

| Inclusion | Exclusion |
| --- | --- |
| *Population* |  |
| - Patients who have undergone DR screening and are referred to eye care providers for referable DR | - Patients undergoing screening for other eye conditions, not including DR |
| *Intervention* |  |
| - AI-assisted grading of retinal images e.g., IDx-DR, ARDA, EyeArt - Automated and semi-automated screening | - Absence of AI-supported grading of DR - Studies with only non-image-based applications of AI |
| *Comparator* |  |
| - Data on adherence to DR follow-up with manual grading or status quo referral pathway, either in existing literature or stated within article |  |
| *Outcome* |  |
| - Measurement of adherence rates to follow-up after detecting referable DR - Specification of follow-up timeframe appropriate for local health system | - Studies which do not report adherence to follow-up as an outcome measure e.g., diagnostic accuracy studies, health economic analyses |
| *Study Characteristics* |  |
| - Randomised controlled trials - Non-randomised clinical studies - Cohort studies - Cross-sectional studies | - Non-primary research e.g., editorials, protocols, trial registrations, matters arising - Review articles - Conference abstracts - Health economic analyses - Articles not written in English |

**Supplementary Table 3: Search strategy across included databases**

|  | **Search Syntax** | | | | |
| --- | --- | --- | --- | --- | --- |
| **Concept** | **MEDLINE** | **Embase** | **Cochrane Library** | **Scopus** | **Web of Science** |
| **Artificial Intelligence** | **Search 1** – Artificial Intelligence/ OR Neural Networks, Computer/ OR exp Machine Learning/ OR Image Processing, Computer-Assisted/    **Search 2** – (“Artificial intelligence” OR automated OR "autonomous AI" OR "computer based analysis" OR "convolutional neural network" OR “deep learning” OR “machine learning”).ti,ab. | **Search 1** – Artificial intelligence/ OR convolutional neural network/ OR machine learning/ OR deep learning/    **Search 2** – (“Artificial intelligence” OR automated OR “autonomous AI” OR “computer based analysis” OR “convolutional neural network” OR “deep learning” OR “machine learning”).ti,ab. | **Search 1** – MeSH descriptor: [Artificial Intelligence] this term only OR MeSH descriptor: [Neural Networks, Computer] this term only OR MeSH descriptor: [Machine Learning] explode all trees OR MeSH descriptor: [Image Processing, Computer-Assisted] this term only  **Search 2** – (“artificial intelligence” OR automated OR "autonomous AI" OR "computer based analysis" OR "convolutional neural network" OR “deep learning” OR “machine learning”):ti,ab | **Search 1 –** TITLE-ABS(“artificial intelligence” OR Automated OR "autonomous AI" OR  "computer based analysis" OR "convolutional neural network" OR “deep learning” OR “machine learning”) | **Search 1 –** TS=(“Artificial intelligence” OR automated OR "autonomous AI" OR "computer based analysis" OR "convolutional neural network" OR “deep learning” OR “machine learning”) |
|  | AND | AND | AND | AND | AND |
| **Adherence to Follow-Up** | **Search 1** – “Treatment Adherence and Compliance”/    **Search 2** – (Adherence OR "follow up" OR referral* OR attend* OR complian* or specialis*).ti,ab. | **Search 1** – Follow up/  **Search 2** – (Adherence OR “follow up” OR referral* OR attend* OR complian* OR specialis*).ti,ab. | **Search 1** – MeSH descriptor: [Treatment Adherence and Compliance] this term only  **Search 2** – (adherence OR "follow up" OR referral* OR attend* OR complian* OR specialis*):ti,ab | **Search 1 –** TITLE-ABS(Adherence OR "follow up" OR referral* or attend* or complian* or specialis*) | **Search 1 –** TS=(Adherence OR "follow up" OR "referral*" OR attend* OR complian* OR specialis*) |
|  | AND | AND | AND | AND | AND |
| **Ophthalmology** | **Search 1** – Diabetic Retinopathy/    **Search 2** – ("Diabetic eye*" OR “diabetic retinopathy”).ti,ab. | **Search 1** – Exp diabetic eye disease/    **Search 2** – (“Diabetic eye*” OR “diabetic retinopathy”).ti,ab. | **Search 1** – MeSH descriptor: [Diabetic Retinopathy] this term only  **Search 2** – ("diabetic NEXT eye* OR "diabetic retinopathy):ti,ab | **Search 1 –** TITLE-ABS(  "Diabetic eye*" OR "diabetic retinopathy") | **Search 1 –** TS=("Diabetic eye*" OR “diabetic retinopathy”) |

**Supplementary Table 4: Details of studies’ interventions and comparators**

| **Author (citation)** | **Study Design** | **Intervention (AI)** | **Comparator** |
| --- | --- | --- | --- |
| Wolf et al. ^1^ | Parallel group randomised controlled trial | **Autonomous:** Patients referred by AI | **Universal referral:** All patients with diabetes referred with educational intervention |
| Mathenge et al. ^2^ | Parallel group randomised controlled trial | **Autonomous:** Patients referred by AI | **Manual grading:** Patients referred by asynchronous telehealth (3-5-day results delay) |
| Liu et al. ^7^ | Prospective cohort study, two-arm sequential | **Autonomous:** Patients referred by AI | **Universal referral:** All patients with diabetes referred and scheduled within 2 weeks of screen |
| Li et al. ^3^ | Prospective cohort study with historical comparator | **Semi-autonomous:** Patients referred by PCPs augmented with AI + LLM output | **Manual grading:** Patients referred by primary care provider |
| Dow et al. ^5^ | Retrospective cohort study | **Autonomous:** Patient referred by AI   - 0-12-hour delay for results | **Manual grading:** Patients referred by asynchronous telehealth (1–5-day results delay)  **Semi-autonomous:** Patients referred by asynchronous telehealth (1-5-day results delay)   - only if deemed ungradable by AI |
| Chotcomwongse et al. ^6^ | Prospective cohort study, two-arm alternating | **Autonomous:** Patients referred by AI | **Manual grading:** Patients referred by trained nurse |

**Supplementary Table 5: RoB-2 Risk of Bias Assessment for RCTs**

**
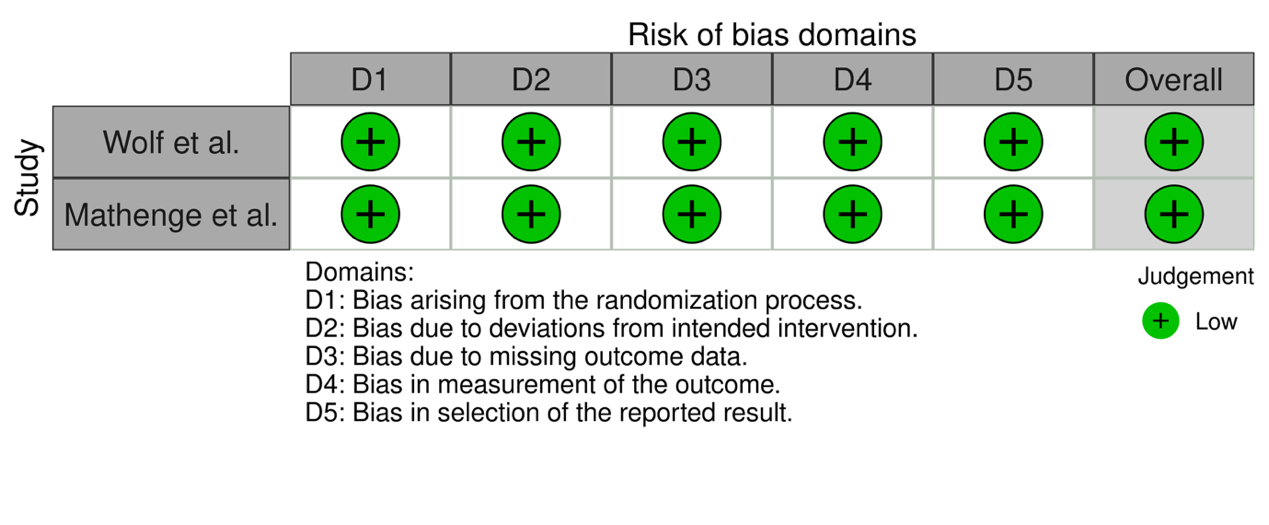
**

**Supplementary Table 6: ROBINS-I Risk of Bias Assessment for observational studies**


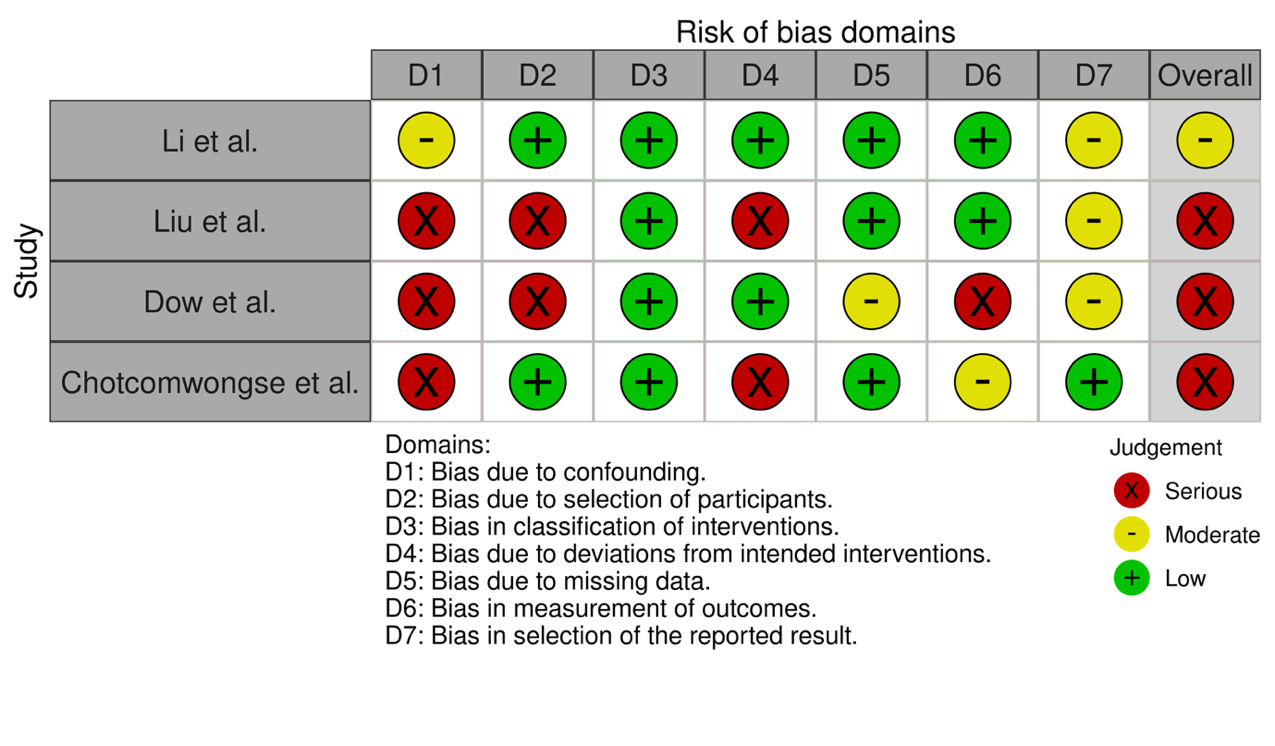


**Supplementary Table 7: Summary of findings for studies reporting data on DR referral uptake**

| **No. of Studies** | **Study Design(s)** | **Risk of Bias** | **Inconsistency** | **Indirectness** | **Imprecision** | **Publication Bias** | **No. of Patients** | **Effect** | **Certainty** |
| --- | --- | --- | --- | --- | --- | --- | --- | --- | --- |
| 6 | 2 RCTs  4 observational | **Not Serious** | **Serious** | **Not Serious** | **Not Serious** | **Serious** | **AI-assisted screening:** 889  **Manual grading:** 1642 | **1.89 (95% CI, 1.175; 3.030)** | **++ Low** |

**Supplementary Table 8: Co-interventions of included studies**

| **Author (citation)** | **Co-intervention** | | | **Comparator Referral Uptake (%)** | **AI Referral Uptake (%)** | **Absolute Effect Size of AI (%)** | **Relative Risk of Referral Uptake** |
| --- | --- | --- | --- | --- | --- | --- | --- |
|  | **Education (inc. grading report)** | **Text reminders** | **Scheduling support** |  |  |  |  |
| Wolf et al. ^1^ |  |  |  | 22.0% | 64.0% | +42.0% | 2.91 |
| Liu et al. ^7^ |  |  |  | 18.7% | 55.4% | +36.7% | 2.96 |
| Dow et al. ^5^ |  |  |  | 12.0% | 35.5% | +23.5% | 2.96 |
| Li et al. ^3^ |  |  |  | 58.4% | 77.8% | +19.4% | 1.33 |
| Mathenge et al. ^2^ |  |  |  | 39.6% | 51.5% | +11.9% | 1.30 |
| Chotcomwongse et al. ^6^ |  |  |  | 77.3% | 89.1% | +11.8% | 1.15 |

**Supplementary Table 9: Referral uptake in order of follow-up timeframe**

| **Author (citation)** | **Study Period of Follow-up** | **Risk of Referral Uptake** | | **AI Time to Follow-up** |
| --- | --- | --- | --- | --- |
|  |  | **Risk with [comparator]** | **Risk with [intervention]** |  |
| Liu et al. Prospective Cohort Study ^7^* | 12 months | 18.7% | 55.4% | 98.0 days to follow-up appointment |
| Wolf et al. ACCESS RCT ^1^* | 6 months | 22.0% | 64.0% | Not discussed |
| Mathenge et al. RAIDERS RCT ^2^* | 90 days | 39.6% | 51.5% | 6.6 ± 7.4 days to “seek” treatment (median 4.0, range 0-30) |
| Dow et al. Retrospective Cohort Study ^5^* | 90 days | 12.0% | 35.5% | Not discussed |
| Li et al. Prospective Cohort Study ^3^* | 2 weeks | 58.4% | 77.8% | 4 days to schedule follow-up (median); IQR 3-5 |
| Chotcomwongse et al. Prospective Cohort Study ^6^* | Unspecified | 77.3% | 89.1% | Not discussed |

**Supplementary Table 10: Excluded studies via full text screening**

| Manuscript title (doi) | Reason for exclusion |
| --- | --- |
| Mitigation of AI adoption bias through an improved autonomous AI system for diabetic retinal disease (https://dx.doi.org/10.1038/s41746-024-01389-x) | Wrong outcomes |
| Autonomous artificial intelligence increases real-world specialist clinic productivity in a cluster-randomised trial (https://dx.doi.org/10.1038/s41746-023-00931-7) | Wrong outcomes |
| Implementation of artificial intelligence-based diabetic retinopathy screening in a tertiary care hospital in Quebec: Prospective validation study (https://dx.doi.org/10.2196/59867) | Wrong outcomes |
| Planning an artificial intelligence-based diabetic retinopathy screening program using human-centred design (https:/dx.doi.org/10.3389/fmed.2023.1198228) | Wrong study design |
| Risk stratification for diabetic retinopathy screening order using deep learning: A multicentre prospective study (https://dx.doi.org/10.1167/tvst.12.12.11) | Wrong intervention |
| Effectiveness of artificial intelligence screening in preventing vision loss from diabetes: A policy model (https://dx.doi.org/10.1038/s41746-023-00785-z) | Wrong outcomes |
| Integration of artificial intelligence into a telemedicine-based diabetic retinopathy screening program (doi not available) | Abstract only |
| A study to evaluate the impact of a software to detect eye diseases (Google-ARDA) among people with type 2 diabetes in India (doi not available) | Wrong study design |
| Detection rate of diabetic retinopathy before and after implementation of autonomous AI-based fundus photograph analysis in a resource-limited area in Belize (https://dx.doi.org/10.2147/opth.s490473) | Wrong outcomes |
| Cost-effectiveness of primary care-based non-mydriatic fundus photography with automated retinal image analysis screening among low-income patients with diabetes (doi not available) | Abstract only |
| Five-year cost-effectiveness modeling of primary care-based nonmydriatic automated retinal image analysis screening among low-income patients with diabetes (https://dx.doi.org/10.1177/1932296820967011) | Wrong study design |
| Implementing autonomous AI in a federally qualified health centre (FQHC) for the detection of diabetic retinopathy improved access to care: A pre-post comparison in Southeastern U.S (https://dx.doi.org/10.2337/db22-69-OR) | Wrong study design |
| Autonomous artificial intelligence for diabetic eye disease increases access and health equity in underserved populations (https://dx.doi.org/10.1038/s41746-024-01197-3) | Wrong outcomes |
| Autonomous artificial intelligence exams are associated with higher adherence to diabetic retinopathy testing in an integrated healthcare system (doi not available) | Abstract only |
| Use of telemedicine and artificial intelligence to improve the way patients are referred from community optometrists to hospital eye units (doi not available) | Wrong study design |
| Artificial intelligence-supported diabetic retinopathy (a complication of diabetes, caused by high blood sugar levels damaging the back of the eye) screening in Tanzania | Wrong study design |
| Real-world outcomes from artificial intelligence to detect diabetic retinopathy in the primary care setting: 12 month experience (doi not available) | Abstract only |
| Revolutionising diabetic retinopathy screening: Integrating AI-based retinal imaging in primary care (https://dx.doi.org/10.1080/28338073.2024.2437294) | Wrong outcomes |
| Autonomous artificial intelligence (AI) increases health equity for patients who are more at risk for poor visual outcomes due to diabetic eye disease (DED) (doi not available) | Abstract only |
| Longitudinal screening for diabetic retinopathy in a nationwide screening program: Comparing deep learning and human grader (https://dx.doi.org/10.1155/2020/8839376) | Wrong outcomes |
| Artificial intelligence in community-based diabetic retinopathy telemedicine screening in urban China: Cost-effectiveness and cost-utility analyses with real-world data (https://dx.doi.org/10.2196/41624) | Wrong study design |
| Automated diabetic retinopathy screening in the primary care setting improved compliance with follow-up ophthalmic care (https://dx.doi.org/10.2337/db19-604-P) | Abstract only |
| Diabetic retinopathy screening in a primary care setting using non-mydriatic photography and automated retinal image analysis improves compliance with follow-up ophthalmic care (doi not available) | Abstract only |
| Application of artificial intelligence-based dual-modality analysis combining fundus photography and optical coherence tomography in diabetic retinopathy screening in a community hospital (https://dx.doi.org/10.1186/s12938-022-01018-2) | Wrong outcomes |
| Autonomous artificial intelligence (AI) testing for diabetic eye disease (DED) closes care gap and improves health equity on a systems level (https://dx.doi.org/10.2337/db23-261-OR) | Abstract only |
| Feasibility of screening for diabetic retinopathy using artificial intelligence, Brazil (https://dx.doi.org/10.2471/BLT.22.288580) | Wrong outcomes |
| Autonomous artificial intelligence for diabetic eye disease testing improves access and equity in the pediatric and adult populations: The Johns Hopkins Medicine experience (https://dx.doi.org/10.2337/dsi24-0016) | Full-text not available |
| A comprehensive strategy of diabetic retinopathy screening in a public health system: Identifying and overcoming obstacles for implementation (https://dx.doi.org/10.1016/j.deman.2023.100192) | Wrong outcomes |
| Synchronous diagnosis of diabetic retinopathy by a handheld retinal camera, artificial intelligence, and simultaneous specialist confirmation (https://dx.doi.org/10.1016/j.oret.2024.05.009) | Wrong outcomes |
| Follow up metrics in primary care clinics after implementation of an artificial intelligence assisted telemedicine screening program for diabetic retinopathy (doi not available) | Abstract only |
| ACCESS: AI for pediatriC diabetic Eye examS Study (doi not available) | Wrong study design |
| Implementation of an integrated system of artificial intelligence and referral tracking for real-time diabetic retinopathy screening (doi not available) | Wrong study design |
| Diabetic retinopathy screening point-of-care artificial intelligence (doi not available) | Wrong study design |
| Evaluating follow-up metrics in an AI assisted telemedicine screening program for diabetic retinopathy in primary care clinics after hiring a patient care navigator (doi not available) | Abstract only |
| Diabetic retinopathy screening using retinal imaging and automated grading with artificial intelligence in Rwanda – A randomised control trial (doi not available) | Wrong study design |
| Feasibility and patient experience of a pilot artificial intelligence-based diabetic retinopathy screening program in Northern Ontario (https://dx.doi.org/10.1080/09286586.2024.2434738) | No comparator |
| Improving access to eye care through community health screenings using artificial intelligence (https://dx.doi.org/10.1080/09286586.2024.2317838) | Wrong patient population |
| Improving access to eye care through community health screenings using artificial intelligence (https://dx.doi.org/10.1080/09286586.2024.2317838) | Wrong patient population |
| Redesigning clinical pathways for immediate diabetic retinopathy screening results (https://dx.doi.org/10.1056/CAT.21.0096) | Wrong intervention |
| An economic analysis for the use of artificial intelligence in screening for diabetic retinopathy in Trinidad and Tobago (https://dx.doi.org/10.7759/cureus.55745) | Wrong study design |
| Artificial intelligence assisted opportunistic screening for referable diabetic retinopathy: from algorithm to real world application (https://dx.doi.org/10.1111/ceo.13632) | Abstract only |
| Implementation of diabetic retinal screenings at primary care triage improved access to care with minimal burden to the PCP (doi not available) | Abstract only |
| Barriers and determinants of referral adherence in AI-enabled diabetic retinopathy screening for older adults in Northern India during the COVID-10 pandemic: Mixed methods pilot study (https://dx.doi.org/10.2196/67047) | No comparator |
| Application effect analysis of artificial intelligence automatic diagnosis system for diabetic retinopathy in elderly diabetic patients in community and hospital (https://dx.doi.org/10.3760/cma.j.cn511434-20210429-00224) | Full-text not available |
| Cost-utility analysis of deep learning and trained human graders for diabetic retinopathy screening in a nationwide program (https://dx.doi.org/10.1007/s40123-023-00688-y) | Wrong study design |
| Future direction for the deployment of deep learning artificial intelligence: Vision threatening disease detection in underserved communities during COVID-19 (https://dx.doi.org/10.4103/sjopt.sjopt_16_23) | Wrong outcomes |
| Barriers to follow-up specialised eye care following retinopathy identification with traditional vs. autonomous artificial intelligence (AI) camera  (https://dx.doi.org/10.2337/db22-48-OR) | Abstract only |
| Telemedical diabetic retinopathy screening in a primary care setting: Quality of retinal photographs and accuracy of automated image analysis  (https://dx.doi.org/10.1080/09286586.2021.1939886) | Wrong outcomes |
| AI diabetic retinopathy screening in a primary care setting in Rural Maine (https://dx.doi.org/10.1007/s11606-024-09319-z) | No comparator |
| Cost-effectiveness of autonomous point-of-care diabetic retinopathy screening for paediatric patients with diabetes  (https://dx.doi.org/10.1001/jamaophthalmol.2020.3190) | Wrong study design |
| Real-world artificial intelligence-based opportunistic screening for diabetic retinopathy in endocrinology and Indigenous healthcare settings in Australia (https://dx.doi.org/10.1038/s41598-021-94178-5) | No comparator |
| Feasibility and acceptance of artificial intelligence-based diabetic retinopathy screening in Rwanda (https://dx.doi.org/10.1136/bjo-2022-322683) | No comparator |
| The SEE study: Safety, efficacy, and equity of implementing autonomous artificial intelligence for diagnosing diabetic retinopathy in youth  (https://dx.doi.org/10.2337/dc20-1671) | Wrong outcomes |

**Extended Discussion**

*Excluded Studies of Relevance*

Several relevant studies were excluded for specific reasons, despite holding relevance to our review question. Ruamviboonsuk et al. ^8^ in a prospective interventional cohort study in Thailand tracked only a subset of AI-referred patients rather than all of the intervention’s referrals. Wolf et al.'s "SEE study" measured adherence to DR screening guidelines rather than follow-up for referable DR ^9^. Similarly, Huang et al. evaluated screening adherence rates rather than specialist follow-up among those with referable disease ^10^. Health economic analyses were also excluded from this review. Our research team conducted a separate review of health economic evaluations for AI-assisted DR screening and found only two articles from the USA included adherence to follow-up as a parameter in their modelling ^11^. Both studies cited Liu et al. ^4^ to inform their parameter choice ^11^.

Five excluded studies provided data on adherence to DR follow-up after AI screening without direct comparators ^12–15^. Bhambhwani et al. (Canada, n=42 referrals) achieved 76% adherence (32/42) with unspecified follow-up timeframe ^14^. Whitestone et al. (Rwanda, n=197 referrals) demonstrated overall adherence to follow-up as 51.8% (102/197) within 30 days in their prospective cohort study. Chauhan et al. conducted a mixed methods study in India including a prospective cohort study on adherence after AI-enabled DR screening during the COVID-19 pandemic ^13^. Of the 159 referrals, only 23 of patients attended (14.5%) within 30 days. Scheetz et al. also conducted a similarly designed mixed methods study in Australia reporting data on 19 referrals, of whom 5 were reported to have followed up (26.3%) within three months of the referral ^15^. Heuer et al.'s retrospective cohort study in rural Norway reported 74% adherence post-AI implementation and an odds ratio (OR) for DR screening of 2.82 (95% CI, 2.42, 3.27, p<0.001) with a baseline OR of 0.86 compared with a non-AI clinic ^16^.

*Intervention Details*

There was variation across, and within, studies regarding the specific AI algorithm used for screening, retinal camera specifications, algorithmic outputs, application of mydriatic agents, and referral pathways. EyeArt (n=2) ^17^, IDx-DR/LumineticsCore^TM^ (n=2) ^18^, Cybersight AI (n=1) ^19^, and the ARDA system (n=1) were the commercially available algorithms used. Li et al. evaluated a combined image analysis software for DR and large language model, named DeepDR-LLM ^3^. ARDA, EyeArt ^17^, and DeepDR ^3^ provided details on DR grade, whereas IDx-DR/LumineticsCore^TM^ ^18^, and Cybersight AI ^19^ provided a binary output of referral or no referral. All algorithms provided a gradability assessment of the image. For image acquisition, there was marked variation in camera utilisation however Topcon NW-400 and Canon CR-2 were the commonest cameras used.

*Definition of Referable DR*

All but one study ^3^ defined referable DR as moderate NPDR or worse as defined by the International Clinical DR (ICDR) severity scale ^20^. This includes moderate NPDR, severe NPDR and proliferative DR ^20^. Diabetic Macular Edema (DME) also constituted “referable DR” in four of the included studies by Chotcomwongse et al. ^6^, Wolf et al. ^1^, Li et al. ^3^, and Liu et al. ^4^. Ungradable images were also considered referrable in four of the included studies (31,36–38) including Dow et al. if the hybrid workflow after AI screening deemed it ungradable along with AI ^5^. Two studies excluded patients with ungradable images (39,40). Mydriasis was integrated into two studies (37,40). Mathenge et al. from Rwanda used mydriatic agents if an image was deemed ungradable and pupil diameter <2.5mm (40). Chotcomwongse et al. studied the effects of mydriasis in the latter five months of their 8-month study ^6^.

***Time to Follow-up Appointment***

Four studies reported data on time taken for patients to follow-up, generally favouring AI approaches. **Li et al. reported a** median of 4 days for scheduling of follow-up among patients (IQR 3-5) after AI-assisted screening versus 7 days (IQR 6-8) after manual grading (p<0.001) ^3^. Similarly, **Mathenge et al. reported a** mean of 6.6 ± 7.4 days to seeking treatment (median 4.0, range 0-30) for the AI group versus 9.6 ± 5.1 days (median 8.0, range 3-28) for those with delayed human grading ^2^. **Liu et al. also did not have a comparator for this outcome, reporting a** mean of 98 days to follow-up appointment after AI-assisted DR screening ^7^. The clinical significance of these findings is questionable and warrants further study with comparisons and analysis of timing to treatment after receiving a referral for DR.
